# A Standardized *Wedelia chinensis* Extract Overcomes the Feedback Activation of HER2/3 Signaling upon Androgen-Ablation in Prostate Cancer

**DOI:** 10.3389/fphar.2017.00721

**Published:** 2017-10-10

**Authors:** Chin-Hsien Tsai, Sheue-Fen Tzeng, Shih-Chuan Hsieh, Chia-Jui Tsai, Yu-Chih Yang, Mong-Hsun Tsai, Pei-Wen Hsiao

**Affiliations:** ^1^Agricultural Biotechnology Research Center, Academia Sinica, Taipei, Taiwan; ^2^Graduate Institute of Life Sciences, National Defense Medical Center, Taipei, Taiwan; ^3^Institute of Biotechnology, National Taiwan University, Taipei, Taiwan

**Keywords:** prostate cancer, apoptosis, animal models, natural products, HER2, HER3

## Abstract

Crosstalk between the androgen receptor (AR) and other signaling pathways in prostate cancer (PCa) severely affects the therapeutic outcome of hormonal therapy. Although anti-androgen therapy prolongs overall survival in PCa patients, resistance rapidly develops and is often associated with increased AR expression and upregulation of the HER2/3-AKT signaling pathway. However, single agent therapy targeting AR, HER2/3 or AKT usually fails due to the reciprocal feedback loop. Previously, we reported that wedelolactone, apigenin, and luteolin are the active compounds in *Wedelia chinensis* herbal extract, and act synergistically to inhibit the AR activity in PCa. Here, we further demonstrated that an herbal extract of *W. chinensis* (WCE) effectively disrupted the AR, HER2/3, and AKT signaling networks and therefore enhanced the therapeutic efficacy of androgen ablation in PCa. Furthermore, WCE remained effective in suppressing AR and HER2/3 signaling in an *in vivo* adapted castration-resistant PCa (CRPC) LNCaP cell model that was insensitive to androgen withdrawal and second-line antiandrogen, enzalutamide. This study provides preclinical evidence that the use of a defined, single plant-derived extract can augment the therapeutic efficacy of castration with significantly prolonged progression-free survival. These data also establish a solid basis for using WCE as a candidate agent in clinical studies.

## Introduction

Androgen receptor plays a critical role in the development and progression of PCa, and therefore androgen-deprivation therapy (ADT) is usually the first-line treatment for metastatic disease. Androgens bind and activate AR transcription factor to regulate many signaling pathways for PCa cell viability, proliferation and invasion (Hara et al., 2008; Snoek et al., 2009). Although ADT is usually initially effective, after 2–3 years many patients develop CRPCwhich is ultimately lethal. Clinical evidence has indicated that AR amplification is a frequent aberration in 60% patients with metastatic CRPC (Robinson et al., 2015). Progression to CRPC is believed to involve the reprogramming of the AR transcriptional landscape and selection pressure for cells to maintain AR activity even at lower concentrations of circulating androgens (Sharma et al., 2013). AR signaling can be maintained through gene amplification (Visakorpi et al., 1995), activating mutations (Steinkamp et al., 2009), AR splice variants (Cao et al., 2016), aberrations in expression levels of AR co-regulators (Heemers et al., 2009), or crosstalk with other oncogenic signaling pathways (Zhu and Kyprianou, 2008). Therefore, adjuvant therapy that targets other essential survival pathways and causes apoptosis during ADT would impede the development of CRPC.

Altered PI3K signaling including PTEN loss is well characterized in PCa with 42% in primary tumors and 100% in metastases (Taylor et al., 2010). Activation of PI3K-AKT signaling may play a critical role, allowing PCa cells to survive in a low-androgen milieu (Majumdar et al., 2007). The ErbB family receptors, including EGFR (ErbB1), HER2 (ErbB2), HER3 and HER4 are most frequently implicated in neoplasia and are known to turn on the PI3K/AKT pathway to regulate cell proliferation, survival, angiogenesis, migration and invasion (Arteaga and Engelman, 2014). The physiological significance of HER2 and HER3 in CRPC is supported by studies showing elevated expression of HER2 and HER3 in CRPC clinical samples (Signoretti et al., 2000; Shi et al., 2001; Chen et al., 2010). It is known that heterodimerization of HER2 with HER3 can mediate AR function at low concentrations of androgen (Mellinghoff et al., 2004). Accordingly, several dual EGFR/HER2 inhibitors, such as lapatinib, have been shown effective against PCa cells in preclinical assays (Gregory et al., 2005; Gravina et al., 2011). However, clinical trials of ErbB2-targeted therapies turned out to be lack of efficacy in patients with either hormone naïve PCa or CRPC (Ziada et al., 2004; de Bono et al., 2007; Sridhar et al., 2010; Whang et al., 2013). In preclinical studies, one of the reasons suggested for this ineffectiveness was that EGFR kinase inhibition rapidly switched on the ErbB signaling network by overexpression of alternative ErbB receptors and ligands to compensate the EGFR/HER2-directed inhibition (Jain et al., 2010; Carrion-Salip et al., 2012). On the other hand, cooperation of AR signaling and ErbB receptors might also cause failure of EGFR/HER2-targeted kinase inhibitors. Studies in cell lines and xenograft models have indicated that ADT can upregulate HER2 and HER3 expression, resulting in the restoration of AR activity and tumor growth in CRPC, while combination with inhibitors against the ErbBs-PI3K-AKT axis can significantly boost the therapeutic effect of ADT (Chen et al., 2011; Thomas et al., 2013; Shiota et al., 2015; Gao et al., 2016). The pathological analysis of PCa specimens showed that activation of HER2/HER3 was increased in association with nuclear AR expression during ADT and remained active in CRPC (Gao et al., 2016). Therefore, it is more reasonable to combine ErbB inhibitor with ADT at an early stage to prevent the progression of CRPC in spite of the risk of additional toxicities.

Herbal medicine has multiple components and is regarded as a less toxic intervention that may exhibit synergistic/additive actions as a monotherapy or in combination with chemotherapy. Dietary factors may influence the biological processes related to prostate carcinogenesis; moreover, epidemiological studies suggest that high consumption of fruits and vegetables is associated with a lower risk of PCa (Cohen et al., 2000; Kolonel et al., 2000; Lin et al., 2015). Given that PCa is a slow-growing disease, but potentially fatal, chemoprevention or adjuvant therapy by using herbal medicine through dietary intervention appears to be a practical and encouraging approach. Previously, we showed that wedelolactone, apigenin and luteolin are highly enriched in the ethanolic extract of *Wedelia chinensis* and exhibit anti-tumor function by inhibiting AR activity and clonogenic growth in PCa cells (Lin et al., 2007). Furthermore, we standardized the preparation of this herbal extract by applying one-step purification of crude herbal extract using column chromatography to enrich the content of wedelolactone, apigenin, and luteolin to ∼70% and by lot-by-lot characterization of the enriched extract by chemical analysis and biological activity using *in vitro* PSA-reporter assay and *in vivo* tumor xenograft assay. The qualified herbal preparation was named *W. chinensis* extract (WCE) (Tsai et al., 2015). In addition, we demonstrated that the benefit of WCE arises from synergistic suppression of the proliferation of AR-expressing PCa cells with prolonged half-lives of active compounds in blood circulation (Tsai et al., 2009, 2015). Previous evidence suggested the potential anti-cancer activity of luteolin and apigenin by downregulating the AR and AKT signaling in PCa cells (Gupta et al., 2002; Chiu and Lin, 2008; Kaur et al., 2008; Shukla et al., 2014). And, wedelolactone also inhibits AR activity by suppressing IKKα which phosphorylates AR to facilitate the AR nuclear translocation and transcriptional activity (Jain et al., 2012). However, AR suppression was shown inducing HER2/3 reactivation and leading to the eventual failure of castration therapy. Therefore, in this study we examined whether a combination of standardized WCE with ADT may enhance the therapeutic efficacy of ADT in PCa or impede the progression of CRPC. This study demonstrates that WCE simultaneously reduced the levels of AR, HER2/3 and AKT activation to intensify the effect of castration and inhibit the progression of CRPC.

## Materials and Methods

### Chemical Reagents and Antibodies

Antibodies to HER2, HER3, AKT1, phospho-AKT1 (S473) and PARP1 were purchased from Cell Signaling (Danvers, MA, United States). Antibodies to cytokeratin-18, phosphor HER3, Ki-67 and GADPH were purchased from Abcam (San Francisco, CA, United States). Anti-pAKT (S473) for IHC staining was from GeneTex (Hsinchu, Taiwan). AR antibody was from Santa Cruz Biotechnology (Dallas, TX, United States). Antibodies to phoso-HER2 and cleaved caspase 3 came from Merck Millipore (Taipei, Taiwan).

### Cell Line and Cell Culture

PC-3, DU145, 22Rv1 and LNCaP cell lines were obtained from American Type Culture Collection (ATCC, Manassas, VA, United States). All cell lines were authenticated by comparing them with the ATCC database of short tandem repeat DNA profiles. For *in vivo* measurement of tumor growth, the cell lines were stably transfected with firefly luciferase luc2 of pGL4 (Promega, Madison, WI, United States) driven by a hybrid EF1α/eIF4g promoter through lentivirus infection (Hsu et al., 2012). All cell lines were cultured in RPMI-1640 (Thermo Fisher Scientific, MA, United States) supplemented with 2 mM glutamine, 1 mM sodium pyruvate and 10% fetal bovine serum (Thermo Fisher Scientific). Cells were maintained at 37°C in a humidified atmosphere containing 5% CO_2_.

### Preparation of *Wedelia chinensis* Extract

The plant materials are cultivated in a greenhouse in Annan District, Tainan City, Taiwan and authentication of the *W. chinensis* plant was performed based on sequencing results of the internal transcribed spacer DNA sequence (ITS) analysis compared with the reference in NCBI databank (Accession: AY947415.1). The whole fresh plants were air-dried, ground and extracted by immersion with ethanol. After condensing, the ethanolic extract was acid-hydrolyzed with HCl at pH 2.0, at 80°C for 30 min to increase aglycone flavonoid content, then neutralized with NaOH and applied to flash LC with a C18 column (SNAP 400 KP-C18-HS Column, Biotage, Uppsala, Sweden) to separate the extract into fractions. To assure consistent WCE quality, the chemical profiles of all WCE lots were analyzed by HPLC-CAD, quantified by triple quadruple LC-MS for contents of wedelolactone, apigenin and luteolin, and analyzed by PSA reporter assay for AR-inhibitory activity.

### Real-Time PCR and Cell Growth Assay

*Wedelia chinensis* was dissolved in DMSO for *in vitro* studies. For *in vitro* mimic of the castration effect, cells were incubated in medium containing 10% CD-FBS with indicated treatments of vehicle control, 5α-dihydrotestosterone (DHT), or WCE for 2 days. After treatment, cell lysates were prepared by lysis with RIPA buffer supplemented with protease and phosphatase inhibitor cocktails (Thermo Fisher Scientific) for immunoblotting. A quantitative polymerase chain reaction (qPCR) analysis was conducted using a Thermo Fisher scientific SYBR Master Mix and an ABI 7500 real-time PCR system. Raw counts were normalized to GAPDH (Δ*C*t) and values are presented as fold change relative to vehicle-treated control (2^-ΔΔCt^). For cell growth assay, 5000 cells were seeded in 96-well plates overnight and treated with different concentrations of WCE for 4 days. The readout of cell number was measured by CyQUANT Direct Cell Proliferation Assay (Thermo Fisher Scientific).

### Luciferase Reporter Assay

For luciferase activity, 22Rv1/103E cells (2 × 10^4^) that were stable clone transfected with PSA promoter-Luciferase gene were grown on 96-well plates with RPMI-1640 medium containing 10% CD-FBS for 18 h. The culture was refreshed with the same medium containing treatments as indicated in the figures and grown for another 18 h. Cells were lysed by passive lysis buffer (Promega) and luciferase assay was performed by luciferase assay system (Promega). Luciferase activity was measured by VICTOR^3^ counter (PerkinElmer, Taipei, Taiwan) and data were normalized to lysate protein as measured by Coomassie (Bradford) Protein Assay Kit (Thermo Fisher Scientific). Inhibition of AR by each treatment was calculated by relative luciferase activity induced by 10 nM DHT as 0% inhibition and vehicle as 100% inhibition.

### *In Vivo* Tumor Xenograft Experiments

Athymic nude mice (6 weeks old) were obtained from the National Laboratory Animal Center (Taiwan) and all animal work was conducted in accordance with a protocol approved by the Institutional Animal Care and Use Committee, Academia Sinica. For orthotopic implantation, male mice were anesthetized by 2.5% isoflurane and 2 × 10^5^ cancer cells in 20 μL of DPBS were injected into the anterior prostate by using a 27 gauge needle fitted on a 50 μL Hamilton syringe. After 1 week of tumor establishment, WCE was administered through gavage at a dosage of 10 mg/kg. For the *in vivo* bioluminescence imaging (BLI) of tumors, mice received D-luciferin at 150 mg/kg by intraperitoneal injection at 10 min prior to imaging and tumor growth was monitored once per week. For *ex vivo* BLI of lung tissues, mice were sacrificed by CO_2_ overdose and lung tissues were removed, immersed in DPBS and imaged for 10 s. The images were acquired with a Xenogen IVIS 50 Imaging System and quantitatively analyzed with Living Image 2.50 software (PerkinElmer).

### Immunochemistry Staining

The formalin-fixed, paraffin-embedded tissue sections were deparaffinized and hydrated in a series of graded alcohol to water. After antigen retrieval, tissue sections were incubated with specific antibodies for 1 h at room temperature. PromARK micro-polymer detection systems (Biocare Medical, Concord, CA, United States) were used to detect the primary antibodies. All images were captured with a Zeiss AxioCam HRc camera attached to a Zeiss AxioImager.Z1 microscope (Munich, Germany). For determination of positive cells, the random five fields per tumor sections (*n* = 6) were examined following staining.

### Statistics

The median inhibition concentration (IC_50_) values of PSA reporter assay were calculated using GraphPad Prism 6.0 (GraphPad Software, La Jolla, CA, United States) using non-linear regression. The significant differences between the groups were compared with Student’s *t*-test (two-tailed) by the same software. A *P-*value of less than 0.05 was considered statistically significant. All values presented in the study were expressed as means with standard error (SEM).

## Results

### Effect of WCE on Cell Cycle Progression and AR Expression in Prostate Cancer Cells

**FIGURE 1:**
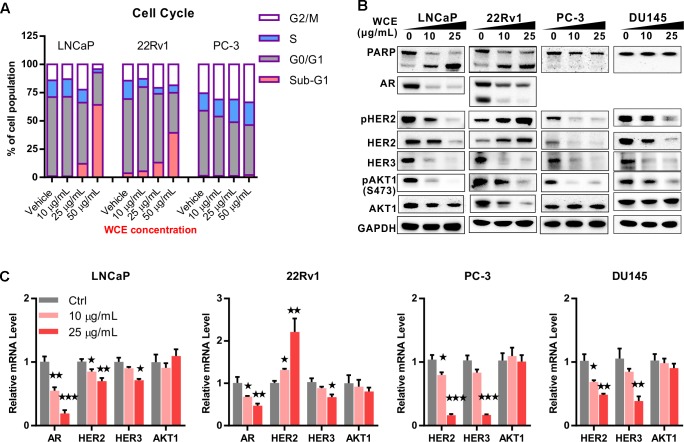
*In vitro* effect of *Wedelia chinensis* (WCE) on cell cycle distribution and androgen receptor (AR) signaling in PCa cell lines. **(A)** The cell cycle profile in vehicle and WCE treated PCa cell lines were determined by staining DNA content and analyzing in flow cytometry. **(B)** Dose-dependent effects of WCE on the expression of PARP, AR, AKT, pAKT1 (S473), HER2, pHER2 (Y1248), and HER3 in LNCaP, 22Rv1, PC-3, and DU145 cells. Cells were grown in medium with 10% FBS for 24 h and treated as marked for another 24 h. **(C)** Effect of WCE on mRNA levels of AR, HER2, HER3, and AKT1 were determined by quantitative real time-PCR. Data are expressed as mean ± SEM. ^∗^*P* ≤ 0.05, ^∗∗^*P* ≤ 0.01, ^∗∗∗^*P* ≤ 0.001 (*t*-test, two-tail).

**FIGURE 2:**
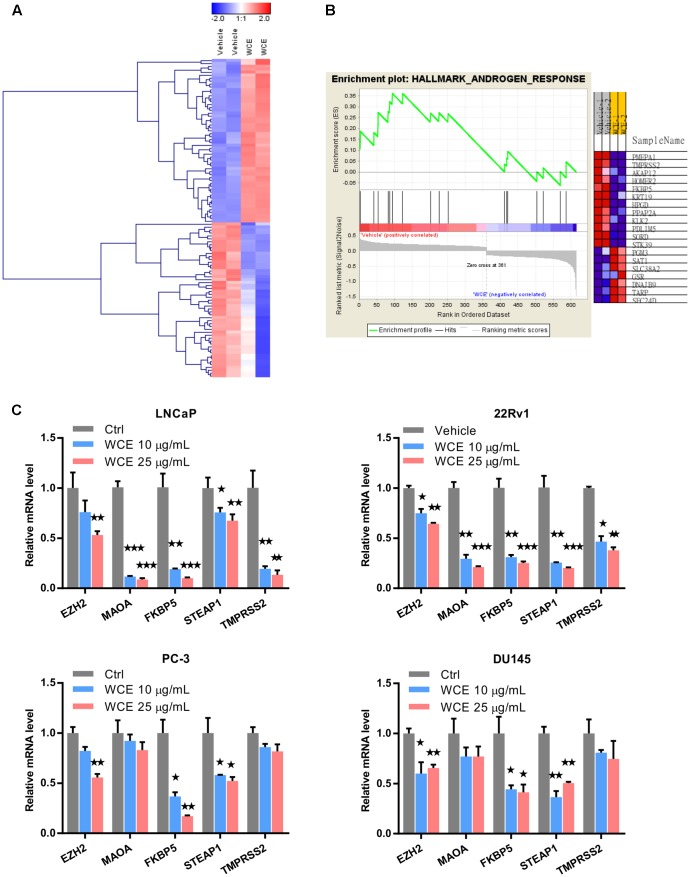
Genes differentially expressed in WCE-treated 22Rv1 cells. **(A)** Clustering of genes differentially expressed in 22Rv1 cells treated with WCE (10 μg/mL) vs. vehicle (DMSO) and cultured for 24 h. The initial analysis was performed on the Illumina microarray platform. **(B)** GSEA were performed with hallmark gene sets of Molecular Signatures Database. Heat map of androgen response gene set representing changes in mRNA level corresponding to vehicle or WCE (10 μg/mL) in 22Rv1 cells (*p*-value = 0.022, FDR *q*-value = 0.108). **(C)** Expression levels of EZH2, MAOA, FKBP5, STEAP1, and TMPRSS2 in LNCaP, 22Rv1, PC-3 and DU145 cells were analyzed by qRT-PCR following indicated treatments. Data are expressed as mean ± SEM. ^∗^*P* ≤ 0.05, ^∗∗^*P* ≤ 0.01, ^∗∗∗^*P* ≤ 0.001 (*t*-test, two-tail).

To evaluate the response of PCa to WCE treatment, we first measured the cell cycle distribution following WCE treatment in three different PCa cell lines: AR-positive, androgen-dependent LNCaP, AR-positive but castration-resistant 22Rv1, and AR-negative, hormone-refractory PC-3. After 24 h incubation with WCE, low-dose WCE (25 μg/mL) induced apoptosis in LNCaP and 22Rv1 cells with 12% and 13% increases, respectively, in the sub-G1 population, while causing cell cycle arrest in the G2/M phase in PC-3 cells (**Figure 1A**). Furthermore, high-dose of WCE (50 μg/mL) induced a 64 and 39% increase, respectively, in the sub-G1 fraction in LNCaP and 22Rv1cells. In apoptotic cells, caspases cleave PARP1 (110-kDa) into 86- and 24-kDa fragments. Immunoblotting of PARP1 showed that the WCE treatment caused a dose-dependent rise and fall of the cleaved 86-kDa fragment and intact PARP1 in 22Rv1 and LNCaP, not PC-3 or DU145 cells (**Figure 1B**). These results suggest that WCE induced caspase-dependent apoptosis in AR-positive PCa cells, not in AR-negative PCa cells. Due to the promising suppression in AR-positive PCa cells, we next determined the WCE effect on AR expression in LNCaP and 22Rv1 cells and found that WCE significantly inhibited the AR expression in PCa cells (**Figure 1B**). As determined by qRT-PCR analysis, the WCE effect on AR was at least in part due to a decrease in AR expression at the transcript and protein levels in PCa cells (**Figure 1C**). Our analysis of WCE responsive genes in 22Rv1 cells using microarray and Gene Set Enrichment Analysis (GSEA) identified a group of androgen responsive genes (**Figures 2A,B** and **Supplementary Table S1**). Genes of FKBP5, STEAP1 and TMPRSS2 are AR target genes with functional androgen-responsive elements in their promoters. As analyzed by qRT-PCR, these AR-responsive genes were dose-dependently downregulated by WCE in both 22Rv1 and LNCaP cells (**Figure 2C**). EZH2 and monoamine oxidase A (MAOA) were correlated with worse clinical outcomes in PCa patients (Xu et al., 2012; Wu et al., 2014). Here, we found that WCE treatment downregulated the mRNA expression of EZH2 and MAOA in AR-positive PCa cells (**Figures 2A,C**). Interestingly, the WCE regulated genes are also downregulated in AR-negative PCa cells except MAOA and TMPRSS2 did not reach statistical significance. It is worth noting that the truncated AR arising from alternative splicing in 22Rv1 was also downregulated by WCE treatment (**Figure 1B**). The C-terminally truncated AR protein lacking the ligand-binding domain (ΔLBD) was constitutively nuclear and actively bound to DNA independent of androgens, resulting in androgen-independent expression of AR target genes and PCa growth (Tepper et al., 2002; Ceraline et al., 2004). In analyses of benign and cancerous prostate tissue, the AR-ΔLBD isoforms were found frequently expressed in clinical PCa (Libertini et al., 2007). WCE suppressed the expression of the AR transcripts and protein and thus suffocated the AR signaling in castration-resistant 22Rv1 cells and induced apoptosis, suggesting the potential use of WCE in patients with hormone-naïve and CRPC.

### WCE Concurrently Downregulates AR, HER3, and AKT Activities

Previous study has indicated that suppression of AR signaling leads to upregulation of HER2/3 signaling and AKT reactivation, which can stabilize the AR and contribute to the eventual failure of ADT (Gao et al., 2016). To determine the role of WCE in the feedback regulation, we examined the expression of HER2 and HER3 upon WCE treatment. Immunoblotting of HER2 and HER3 showed that increased HER2 but decreased HER3 occurred in 22Rv1 cells following WCE treatment, while HER2, phosphorylated HER2, and HER3 proteins were all decreased in LNCaP, PC-3, and DU145 cells (**Figure 1B**). Downstream of HER2/3, AKT phosphorylation at S473 was suppressed after WCE treatment in all four cell lines (**Figure 1B**). Next, we determined whether the effect is regulated at the transcriptional level. In 22Rv1 cells, HER2 mRNA was dose-dependently upregulated by WCE treatment (**Figure 1C**). This is consistent with previous reports that inhibition of AR transcriptional activity in 22Rv1 cells induced HER2 expression (Pignon et al., 2009). However, the mRNA level of HER2 was lowered in LNCaP, PC-3, DU145 cells following WCE treatment compared to vehicle-treated control cells, suggesting that WCE-mediated HER2 regulation involves mechanisms dependent on cell context but probably independent of AR (**Figure 1C**). WCE-mediated transcription of HER3 in PCa cells is not fully constant with respective HER3 protein level, especially at low dose of WCE treatment, suggesting that posttranslational regulation may also be involved in WCE-mediated HER3 degradation (**Figures 1B,C**). Although WCE treatment raised HER2 expression in 22Rv1 cells, WCE also reduced the AKT expression to impair HER2/3 signaling (**Figure 1B**). The mRNA level of AKT was unaffected by WCE, suggesting translational or posttranslational regulation of WCE-mediated AKT expression was involved (**Figure 1C**). WCE decreased the expression of HER2 and HER3 at the protein and mRNA levels in PCa cell lines except HER2 expression in 22Rv1 cells. Consistently, AR-expressing PCa cell lines were more sensitive to WCE than AR-negative cell lines. Of note, WCE effectively disrupted the crosstalk between the HER2/3-AKT pathway and AR signaling in LNCaP and 22Rv1 cells.

### WCE Sensitizes Androgen-Dependent and Castration-Resistant Cell Lines to Apoptosis Induced by Androgen Withdrawal

**FIGURE 3:**
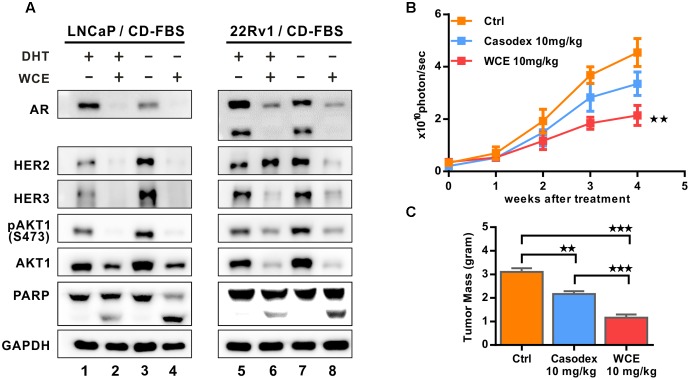
Effect of hormone therapy and WCE on androgen-dependent LNCaP and castration-resistant 22Rv1 cells. **(A)** Immunoblot analysis of HER2, pHER2 (Y1248), HER3, AR, AKT, pAKT1 (S473), and PARP in LNCaP and 22Rv1 cells under WCE, DHT, or combination treatment. Cells were grown in medium with 10% CD-FBS for 24 h to mimic castration and treated as marked for another 24 h. **(B)** *In vivo* tumor growth curve of 22Rv1 cells following treatments of vehicle, 10 mg/kg Casodex, or 10 mg/kg WCE. **(C)** The end-point tumor weight from indicated groups. Data are expressed as mean ± SEM. ^∗∗^*P* ≤ 0.01, ^∗∗∗^*P* ≤ 0.001 (*t*-test, two-tail).

To determine the effect of WCE combined with ADT, cells were grown in a medium stripped by charcoal to mimic ADT, and treated with WCE in the presence or absence of androgen (1 × 10^-9^ mol/L DHT). In androgen-dependent LNCaP cells, lack of androgen significantly decreased the expression of AR protein but increased the expression of HER2, HER3 and downstream AKT phosphorylation without induction of apoptosis, (**Figure 3A**, lane 1 vs. lane 3). Unlike ADT, WCE in the presence of androgen concurrently abolished both AR and HER2/3 signaling and induced apoptosis (**Figure 3A**, 1ane 2 vs. lane 1); WCE combined with ADT further augmented PARP1 cleavage over WCE or ADT alone (**Figure 3A**, lane 4 vs. lanes 2, 3). In castration-resistant 22Rv1 cells, ADT slightly decreased AR expression and resulted in activated HER2-AKT signaling (**Figure 3A**, lane 7 vs. lane 5). Despite the HER2 upregulation by WCE in 22Rv1, WCE decreased the expression of AKT and AR proteins, still leading to cell apoptosis (**Figure 3A**, lane 6 vs. lane 5). Moreover, WCE combined with ADT decreased the expression of AR, HER2 and HER3, enhancing the cell apoptosis (**Figures 3A**, lane 8 vs. lane 6, 7). These effects were next examined *in vivo* using a 22Rv1 orthotopic xenograft model. The anti-androgen drug Casodex moderately delayed the tumor growth leading to 30% lower tumor weight compared to the vehicle control; however, WCE treatment significantly inhibited tumor growth and caused 60% lower tumor weight at end-point (**Figures 3B,C**).

### WCE Complements Castration Therapy in an Androgen-Dependent PCa Model

**FIGURE 4:**
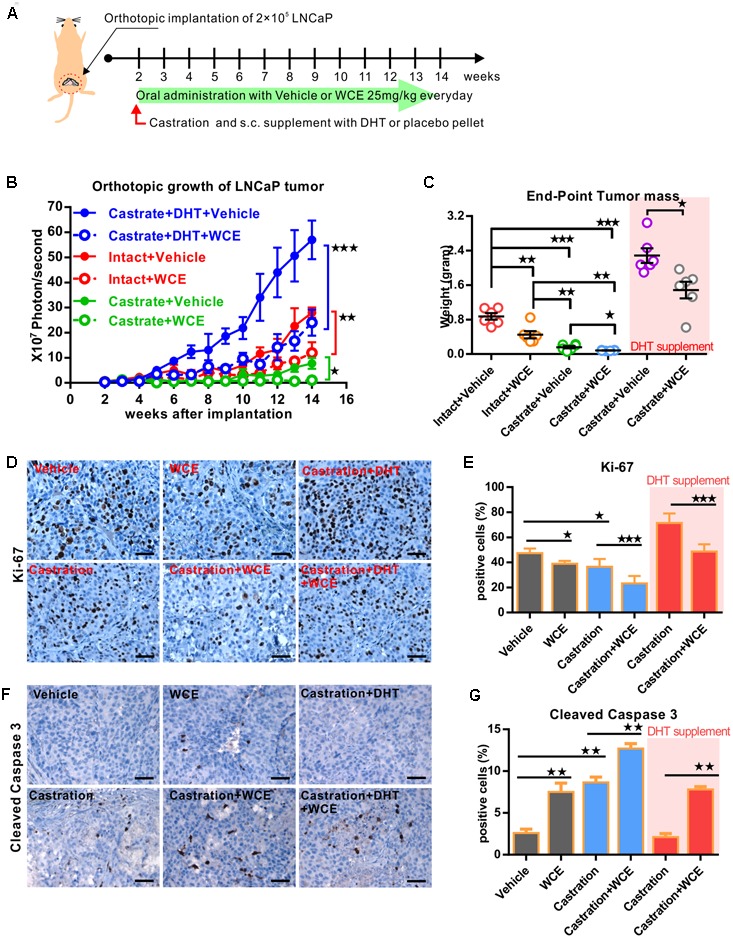
Therapeutic effect of WCE in combination with androgen ablation on an orthotopic xenograft model of hormone-dependent PCa. **(A)** Flow chart for evaluating the *in vivo* effect of WCE on LNCaP orthotopical tumor model. LNCaP-Luc2 cells (2 × 10^5^) were implanted in mouse prostate. After 2 weeks, mice were randomized into six groups: vehicle control, 10 mg/kg of WCE treatment, castration therapy in the presence of androgen pellet or placebo pellet supplement, and combination therapy with or w/o androgen pellet supplement. WCE were orally administered every day for 12 weeks. **(B)** The longitudinal BLI of LNCaP-Luc2 prostate tumor was monitored weekly and is presented as a growth curve (*n* = 6). **(C)** Endpoint mass of primary tumors in each treatment group are presented as a column scatter plot (*n* = 6). Horizontal lines, mean; Bar, SEM. **(D,E)** *In vivo* effects of WCE, castration, or combination on cell proliferation determined by Ki-67 staining. The represented images of Ki-67 staining from indicated groups **(D)**. Ki-67-positive cells are quantified as fractions (%) by averaging readouts from four random fields per tumor section, *n* = 6 **(E)**. **(F,G)** Apoptosis in treated tumors were indicated by cleaved caspase 3 staining. Active caspase 3-positive cells **(F)** in the specimens of primary tumor and calculated as a percentage of total cells counted (*n* = 6, **G**). Data are expressed as mean ± SEM. ^∗^*P* ≤ 0.05, ^∗∗^*P* ≤ 0.01, ^∗∗∗^*P* ≤ 0.001 (*t*-test, two-tail).

**FIGURE 5:**
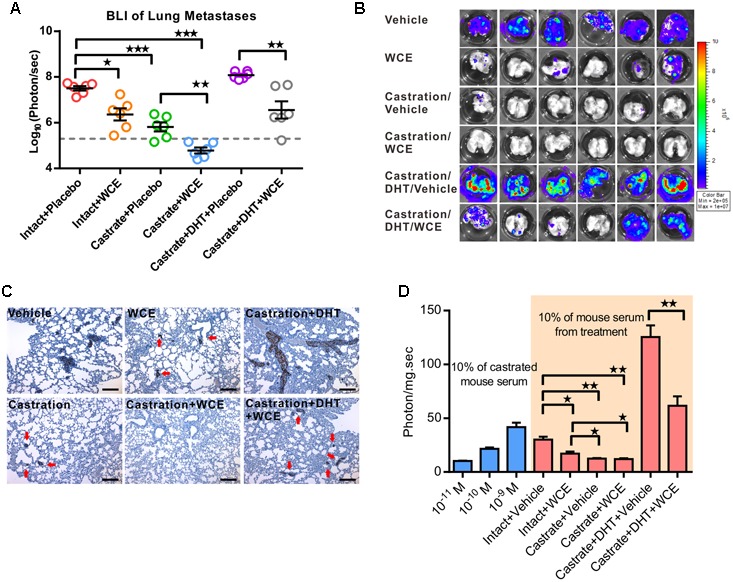
Effect of WCE on *in vivo* metastasis and activity of AR signaling. **(A)** The lung metastasis following treatment was quantified by BLI as photon flux per sec. The results of different treatments are represented as a column scatter plot. Horizontal lines, mean (*n* = 6). **(B)** The *ex vivo* bioluminescent images of the host lungs from representative mice taken at endpoint. **(C)** Lung metastases were identified by IHC staining of human cytokeratin 18. **(D)** Anti-androgen activity of serum samples were analyzed by PSA reporter assay (*n* = 6). Left, indicated concentrations of DHT were added to 10% castrated mouse serum as controls to calibrate androgen levels. Data are expressed as mean ± SEM. ^∗^*P* ≤ 0.05, ^∗∗^*P* ≤ 0.01, ^∗∗∗^*P* ≤ 0.001 (*t*-test, two-tail).

To consider the clinical feasibility of WCE, we investigated the effect of castration in combination with oral WCE treatment in an LNCaP orthotopic xenograft in a nude mouse model (**Figure 4A**). WCE or castration monotherapy markedly reduced tumor growth compared with the vehicle control; and a combination of both therapies further reduced tumor growth (**Figures 4B,C**). Consistent with the tumor growth result, immunohistochemical staining of Ki-67 and cleaved caspase 3 showed that castration or WCE alone inhibited the cell proliferation and induced apoptosis, and combination therapy further enhanced the therapeutic effect (**Figures 4D–G**). While either monotherapy markedly decreased lung metastasis compared with vehicle treatment, combination therapy was also more effective in inhibiting metastasis than either castration or WCE monotherapy (**Figures 5A,B**). Only a few micrometastatic PCa foci were detected in the lungs of the WCE or castration monotherapy groups, and none were detected in the combination group (**Figure 5C**).

To understand whether WCE might affect tumor growth through regulating the physiological levels of androgens, we studied the WCE function under DHT replacement using 12.5 mg of DHT pellet with 60 days release (Innovative Research of America, Sarasota, FL, United States). Despite being used alongside ectopic hormone replacement, WCE remained effective in attenuating tumor growth and metastasis, suggesting that WCE regulated the AR and downstream activities rather than the physiological levels of androgens (**Figures 4B,C**, **5A–C**). To further understand the anti-androgenic activity of WCE in the treated mice, mouse serum containing testosterone, DHT, and WCE compounds was harvested 2 h following the last WCE dose and analyzed by PSA-reporter assay. The active compounds in sera from WCE-treated mice remained functional and inhibited both the physiological and DHT-replaced levels of androgen activity (**Figure 5D**).

**FIGURE 6:**
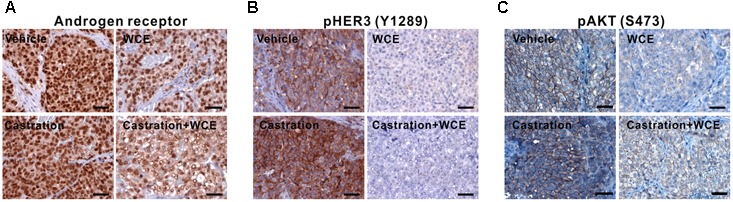
Pathological analyses of LNCaP tumor after the indicated treatments. **(A)** IHC staining of anti-AR antibody in primary LNCaP tumor. **(B)** HER2/3 activity was determined by staining HER2-mediated phosphorylation of HER3 on Y1289. **(C)** AKT activation was examined by anti- phospho-AKT (S473) staining.

Castration treatment for 12 weeks upregulated HER3 phosphorylation (Y1289), AKT phosphorylation (S473) and AR expression in tumors (**Figures 6A–C**). This alteration reflects the transition of androgen-dependent PCa to CRPC and enhancement of cancer survival during ADT by upregulating the AR and HER3 signaling pathways (Gao et al., 2016). However, WCE treatment not only suppressed the AR but also suppressed HER2-dependent phosphorylation of HER3 at Y1289, preventing ADT-induced feedback activation of HER2/HER3 (**Figures 6A,B**). Compared to monotherapy, combination therapy of WCE and ADT showed a more potent therapeutic outcome by suppressing HER3 activation and AR expression. Overall, in contrast with castration, which has unfavorable side effects, WCE treatment decreased the levels of AR, HER2/HER3, and AKT phosphorylation in PCa (**Figures 6A–C**).

### WCE Suppresses the Growth of LNCaP-Derived CRPC Cells

**FIGURE 7:**
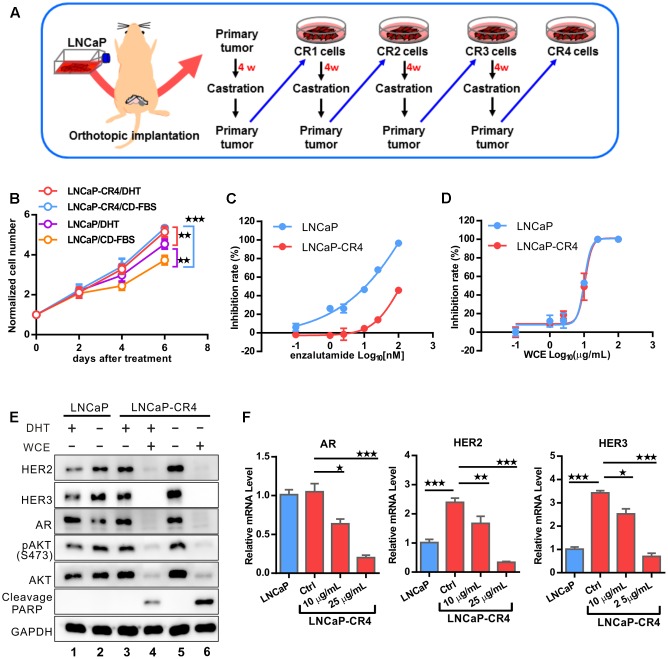
Development of LNCaP CRPC model to evaluate the response to WCE treatment. **(A)** Flowchart showing the development of LNCaP-Luc2-derived CRPC cells. LNCaP-Luc2 cells (2 × 10^5^) were orthotopically injected in mouse prostate. After 4 weeks, mice were castrated and incubated for 7 weeks to allow recurrence of LNCaP tumors. The procedure was repeated three more times to derive LNCaP-CR4 cells. **(B)** The androgen response of LNCaP and LNCaP-CR4 cells were determined by a cell growth curve. Cells were incubated in the androgen-deprived medium with or without 10^-9^ DHT for 6 days. The medium was replaced every 3 days. **(C)** Effect of antiandrogen drug enzalutamide on cell growth of LNCaP and LNCaP-CR4 cells. **(D)** Dose-dependent effect of WCE on cell growth curve. **(E)** Western blot of HER2, HER3, AR, pAKT1, AKT1, and PARP in LNCaP-CR4 cells following the indicated treatments. **(F)** mRNA levels of AR, HER2 and HER3 of LNCaP and LNCaP-CR4 in different treatment groups were determined in qRT-PCR. Data are expressed as mean ± SEM. ^∗^*P* ≤ 0.05, ^∗∗^*P* ≤ 0.01, ^∗∗∗^*P* ≤ 0.001 (*t*-test, two-tail).

*Wedelia chinensis* not only impaired the AR expression but also the HER2/3-AKT signaling in castration-resistant 22Rv1, suggestion a potential function in the prevention of the development of CRPC. Next, we investigated the effect of WCE on another CRPC model (**Figure 7A**). After LNCaP PCa relapsed from castration in mice, we performed necropsies on the mice and cultured the relapsed LNCaP tumor cells, named LNCaP-CR cells. The same procedure was repeated four times and LNCaP-CR4 cells were derived. *In vitro*, growth of LNCaP-CR4 was not affected by ADT in the medium, whereas growth of the parental LNCaP cells was inhibited by ADT (**Figure 7B**). Upon treatment with enzalutamide, a second-line anti-androgen, LNCaP-CR4 cells were ∼10-fold more resistant than the parental LNCaP cells (**Figure 7C**). Therefore, LNCaP-CR4 cells represented a state of CRPC. To this end, WCE elicited the same concentration-effect relationship on the cell growth of LNCaP and LNCaP-CR4 cells (**Figure 7D**). In addition, LNCaP-CR4 expressed higher levels of HER2, HER3, and AKT phosphorylation compared with parental LNCaP cells (**Figure 7E**, lane 3 vs. lane 1). Upon ADT, LNCaP-CR4 cells still expressed a high level of AR (**Figure 7E**, lane 3 vs. lane 5), while ADT significantly decreased AR expression in LNCaP cells, (**Figure 7E**, lane 2 vs. lane 1). Activation of HER2, HER3 signaling in LNCaP-CR4 might arise from upregulation of gene transcription while the increased AR expression may be influenced by HER2/3 activation (**Figure 7F**). Even following the failure of ADT, WCE treatment in PCa cells still effectively inhibited the expression of AR, HER2, and HER3 at the protein and mRNA levels, which also resulted in a decrease in AKT phosphorylation and promotion of apoptosis (**Figure 7E**, lane 6 vs. lane 5, and **Figure 7F**). In conclusion, the data suggest that exposure to effective doses of WCE can completely inhibit the cell growth in LNCaP and LNCaP-CR4 cells at least in part due to effective simultaneous suppression of AR and HER2/3.

## Discussion

Our previous study determined that the major active compounds in WCE are wedelolactone, luteolin and apigenin. Wedelolactone is an IKKα/β inhibitor which has been shown to reduce viability of PCa cells via down-regulation of PKCε (Sarveswaran et al., 2012). Apigenin and luteolin are non-toxic non-mutagenic flavonoid with potentials for cancer therapy, and widely distributed in many fruits and vegetables (Duthie and Crozier, 2000; Weng and Yen, 2012). The dose of 10 μg/mL WCE contained about 3.4–4.9 μmol/L apigenin, 7.7–13.3 μmol/L luteolin, and 2.8–4.6 μmol/L wedelolactone (Tsai et al., 2015). In LNCaP cells under androgen-containing conditions, 10 μmol/L luteolin causes 22% AR protein degradation probably through repressing chaperone HSP90, whereas 10 μmol/L apigenin downregulates AR activity without inhibiting the levels of AR mRNA and protein (Gupta et al., 2002; Chiu and Lin, 2008). Nevertheless, 10 μg/mL WCE significantly decreased 45 and 32% mRNA of AR in LNCaP and 22Rv1 cells, respectively.

**FIGURE 8:**
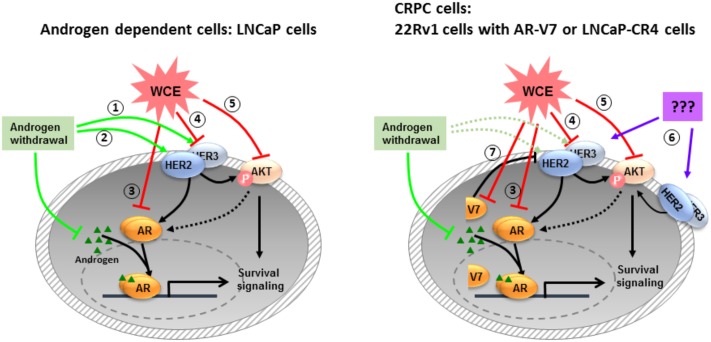
The mechanisms of WCE actions on PCa proposed by this study. In androgen-dependent PCa, androgen withdrawal induces HER2/3 signaling to stabilize AR at low androgen levels (1, 2). WCE inhibits the expression levels of AR (3), HER2/3 (4), and AKT (5) simultaneously to induce cell apoptosis. Most CRPCs express high-level HER2/3 (6), which activates AR signaling through crosstalk signaling. There are also some CRPCs like 22Rv1 cells expressing truncated AR variants that constitutively regulate gene expression for cell growth and survival (7).

In addition, luteolin and apigenin inhibit IGF1R signaling in PCa (Fang et al., 2007; Shukla and Gupta, 2009). However, there is lack of data about the effect of these compounds on HER2/3 signaling in PCa. Heterodimers of EGFR/HER3 and HER2/HER3 are potent pairs for PI3K/AKT-mediated survival signaling. And, a previous study demonstrated that HER3 is required and sufficient to promote the invasion of PCa cells (Soler et al., 2009). Specific inhibitors that disrupt one receptor pair may still allow the signaling to continue through another receptor pair. EGFR and EGFR/HER2 inhibitors, alone or combination with chemotherapeutic agents have not shown the anticipated efficacy in CRPC clinical trials (Ziada et al., 2004; Canil et al., 2005; Nabhan et al., 2009). Therefore, the simultaneous inhibition of these dimers in PCa will leave no dimerization and halt the oncogenic signaling. In androgen-dependent LNCaP cells, HER2 and HER3 expression are negatively regulated by the AR pathway (Pignon et al., 2009; Chen et al., 2010). AR downregulation by WCE supposedly leads to accumulation of HER2 and HER3 proteins (Signoretti et al., 2000; Shi et al., 2001; Chen et al., 2010). Contradictorily, our data showed that WCE treatment resulted in downregulation of HER2, HER3, AR, and AKT phosphorylation, indicating that WCE also regulates the expression of HER2, HER3, and AKT independent of AR (**Figure 1B**). On the other hand, suppression of AR signaling may activate the PI3K/AKT pathway because loss of AR-mediated FKBP5 expression which in turn assists the folding of AKT phosphatase PHLPP would promote AKT activation and the eventual failure of castration therapy (Carver et al., 2011). For hormone-naïve PCa ADT treatment upregulates the transcript and protein levels of HER2 and also increase HER3 protein by decreasing AR-dependent transcriptional upregulation of neuregulin receptor degradation protein-1 (Nrdp1), an E3 ubiquitin ligase that targets HER3 for degradation (Pignon et al., 2009; Chen et al., 2010). Based our data, we proposed a working model in **Figure 8**; ADT treatment increases HER3 and HER2 which activates AR signaling and promotes PCa survival at low levels of androgen. Previous study indicated that luteolin reduced the association between AR and HSP90, causing AR degradation through a proteasome-mediated pathway (Chiu and Lin, 2008; Fu et al., 2012). HSP90 is also required for the stability of AKT and HER2/3 (Basso et al., 2002; Citri et al., 2004). In LNCaP cells, WCE treatment may inhibit HSP90 thus decrease the protein levels of AR, HER2/3, and AKT to prevent ADT-induced HER2/3 upregulation, therefore combination of ADT and WCE significantly advanced the therapeutic effect. Furthermore, wedelolactone also inhibits AR activity by suppressing IKKα which phosphorylates AR to increase nuclear localization of AR and transcriptional activity of AR (Jain et al., 2012). In the CRPC sublines, 22Rv1 and LNCaP-CR4 cells express elevated HER3 level, partially due to the constitutive low expression of Nrdp1 regardless of androgen levels. ADT or suppression of full-length AR in castration-resistant 22Rv1 cells did not significantly regulate the HER2 expression, whereas silencing of the truncated AR in 22Rv1 cells would increase HER2 expression. WCE inhibits the express levels of AR, HER2/3, and AKT in all PCa cell lines. However, in 22Rv1 cells WCE decreased both full-length and truncated ARs, therefore caused a net increase of HER2 protein, its autophosphorylation, and signaling activity. Despite WCE activated HER2, the downstream AKT phosphorylation was counteracted by the WCE inhibition to AKT protein levels, therefore a net decrease of AKT phosphorylation exhibited by WCE treatment.

At early-stage PCa, ADT is effective in hormone-dependent PCa but ADT elicits a marked increase in HER3 protein and activation of downstream signaling, which may be responsible for the oncogenic cell survival and the promotion of CRPC (Chen et al., 2010). This in turn augments AR transcriptional activity and cell proliferation, signaling the reentry of quiescent tumor cells into active cell cycles. The problem with anti-androgens is the eventual development of CRPC. Use of inhibitors of ErbB kinases in patients with CRPC have little effect probably due to incomplete inactivation of AKT signaling, reactivated AR signaling, or both (Chen et al., 2011). Conversely, HER3 downregulation via siRNA suppressed cell viability and impeded CRPC growth (Chen et al., 2010). These studies reveal the significant crosstalk between HER3 and AR and indicate a mechanism by which cells may develop resistance to monotherapy of EGFR/HER2 inhibitor or ADT. WCE is comparable to castration according to our *in vivo* and *in vitro* growth assays (**Figures 3B**, **4B**). Furthermore, *in vitro* growth assay showed WCE has better efficacy than androgen ablation, and WCE treatment significantly induced apoptosis in LNCaP, LNCaP-CR4, and 22Rv1 cells (**Figures 3A**, **7E**). LNCaP cells express full-length AR, whereas 22Rv1 cells express both full-length AR and truncated AR variants encoded by mRNA lacking exons 5–7 (Li et al., 2011; Cao et al., 2014). The C-terminally truncated AR protein lacking the ligand-binding domain (ΔLBD) was constitutively nuclear and actively bound to DNA independent of androgens due to alternative splicing or non-sense mutations of the AR gene, resulting in androgen-independent expression of AR target genes and PCa growth. Higher levels of AR splice variants have been detected in CRPC when compared with hormone-naïve PCa (Zhang et al., 2011). WCE reduces the expression of both full-length AR and truncated AR variants thus affects the endogenous AR signaling in both androgen-dependent LNCaP as well as castration-resistant 22Rv1 cells. This suggests the possibility that WCE may still be effective against clinical CRPC if WCE dosage is high enough.

The most effective therapy is to apply combination therapy at the early stage of PCa to prolong the progression-free survival of ADT. Here, we showed that WCE can concomitantly target HER2/3, AR and AKT to maximize the therapeutic effects of ADT in treating AR-positive PCa without activating the feedback pathways. WCE might inhibit AR gene transcription and thus effectively downregulate full-length and truncated AR. Furthermore, we established castration-resistant LNCaP cells and demonstrated their clinical pathological characteristics, i.e., high levels of AR, HER3 and AKT activities. A further novel finding is that WCE significantly inhibited expression of EZH2 and MAOA. Aberrant overexpression of EZH2 and MAOA correlated with poor prognosis in PCa patients. MAOA has been demonstrated to promote prostate tumorigenesis and cancer metastasis, and this study revealed that WCE inhibited MAOA expression, a promising therapeutic effect (Wu et al., 2014). A previous study indicated that androgen can activate MAOA expression by AR-regulated Sp1 binding to MAOA promoter in human neuroblastoma and glioblastoma cells (Ou et al., 2006). However, the molecular mechanism of WCE-mediated suppression of MAOA and EZH2 expression in PCa needs further investigation.

## Conclusion

Our data also showed that the defined herbal extract WCE can improve the therapeutic outcome of PCa as an add-on to hormonal therapy for androgen-dependent disease and may be effective for use in AR-positive CRPC.

## Author Contributions

C-HT, S-FT, S-CH, C-JT, M-HT, and Y-CY participated in data collection and analysis. C-HT and P-WH participated in the design of the study, data interpretation and the writing of the manuscript.

## Conflict of Interest Statement

The authors declare that the research was conducted in the absence of any commercial or financial relationships that could be construed as a potential conflict of interest.

## Supplementary Material

TABLE S1 WCE-mediated differential genes.

## Abbreviations

AR: androgen receptor
CD-FBS: charcoal-coated dextran-stripped fetal bovine serum
CRPC: castration-resistant prostate cancer
DHT: 5α-dihydrotestosterone
PARP: poly (ADP-ribose) polymerase
PCa: prostate cancer
WCE: *Wedelia chinensis* extract.
